# Dynamic RNA-Seq Study Reveals the Potential Regulators of Seed Germination in *Paris polyphylla* var. *yunnanensis*

**DOI:** 10.3390/plants11182400

**Published:** 2022-09-15

**Authors:** Zhengbin Tang, Jia Zhao, Bin Yang, Shan Sun, Furong Xu, Zhoufei Wang

**Affiliations:** 1 The Laboratory of Seed Science and Technology, Guangdong Key Laboratory of Plant Molecular Breeding, South China Agricultural University, Guangzhou 510642, China; 2 Guangzhou Key Laboratory for Research and Development of Crop Germplasm Resources, Zhongkai University of Agriculture and Engineering, Guangzhou 510225, China; 3 College of Traditional Chinese Medicine, Yunnan University of Chinese Medicine, Kunming 650500, China

**Keywords:** *Paris polyphylla*, seed germination, carbohydrate metabolism, lipid metabolism, signal transduction

## Abstract

*Paris polyphylla* var. *yunnanensis* is an important traditional Chinese medicine, but poor seed germination limits its large-scale artificial cultivation. Thus, it is crucial to understand the regulators of seed germination to obtain clues about how to improve the artificial cultivation of *Paris polyphylla*. In this study, the seeds at three germination stages, including ungerminated seeds (stage 1), germinated seeds with a 0.5 cm radicel length (stage 2), and germinated seeds with a 2.0 cm radicel length (stage 3) after warm stratification (20 °C) for 90 days were used for RNA sequencing. Approximately 220 million clean reads and 447,314 annotated unigenes were obtained during seed germination, of which a total of 4454, 5150, and 1770 differentially expressed genes (DEGs) were identified at stage 1 to stage 2, stage 1 to stage 3, and stage 2 to stage 3, respectively. Encyclopedia of Genes and Genomes (KEGG) analysis revealed that the DEGs were significantly enriched in carbohydrate metabolism, lipid metabolism, signal transduction, and translation. Of them, several genes encoding the glutamate decarboxylase, glutamine synthetase, alpha-galactosidase, auxin-responsive protein IAA30, abscisic-acid-responsive element binding factor, mitogen-activated protein kinase kinase 9/18, and small and large subunit ribosomal proteins were identified as potentially involved in seed germination. The identified genes provide a valuable resource to study the molecular basis of seed germination in *Paris polyphylla* var. *yunnanensis*.

## 1. Introduction

*Paris polyphylla* var. *yunnanensis* (afterward *P*. *polyphylla),* also named ‘Dian Chonglou’ in Chinese, is mainly distributed in Yunnan, Sichuan, and Guizhou provinces in China. The dried rhizomes of *P*. *polyphylla* are a traditional Chinese medicinal herb [1]. Wild *P*. *polyphylla* has been facing extinction due to overharvesting in the past decades, and thus artificial cultivation is the best way to meet commercial demands [2,3]. However, only 40% of seeds can germinate after a long dormancy period (18 months or longer) under natural conditions [4]. Previously, constant exposure to warm conditions (around 20 °C) was known to promote the early germination and relatively rapid radicle growth of *P. polyphylla* [5]. Therefore, understanding the regulators of seed germination during warm stratification will contribute to obtaining clues about how to improve the artificial cultivation of *P*. *polyphylla*.

Seed germination is regulated by the coordination of several complex physiological, biochemical, and molecular processes, such as the mobilization of stored reserves, signaling transduction, and transcription activation [6]. Numerous transcriptomic and proteomic studies have revealed that several regulators of carbohydrate, protein, and lipid metabolisms are involved in seed germination [7,8]. For example, the unigenes involved in the metabolism, such as glycerolipid metabolism, fatty acid degradation, and starch and sucrose metabolism, are differentially expressed during seed germination in *Cinnamomum migao* [8]. To date, several transcriptomic pieces of research have revealed that the genes associated with hormone metabolism and signaling pathways are involved in the seed dormancy of *P. polyphylla* [3,5]. For example, a total of 95 metabolic and 62 signaling genes related to abscisic acid (ABA), gibberellins (GA), auxin, cytokinin, ethylene (ET), brassinosteroids (BR), jasmonic acid (JA), and salicylic acid (SA) have been identified involving in seed dormancy release during warm stratification in *P. polyphylla* [5]. However, the key regulators of carbohydrate and lipid metabolisms involved in seed germination are not yet fully understood in *P*. *polyphylla*.

Abscisic acid (ABA) and gibberellins (GA) are the key internal signals acting antagonistically in seed germination [9]. For example, AtPER1 enhances primary seed dormancy and reduces seed germination by suppressing ABA catabolism and GA biosynthesis in *Arabidopsis* [10]. Rice OsPK5 improves seed germination by influencing glycolytic metabolism and GA/ABA balance [11]. The ABA-INSENSITIVE 5 (ABI5) modulates seed germination via the feedback regulation of PYR/PYL/RCAR ABA receptor genes [12]. CONSTITUTIVE PHOTOMORPHOGENIC 1 promotes seed germination by destabilizing RGA-LIKE 2 in *Arabidopsis* [13]. Moreover, mitogen-activated protein kinase (MAPK) pathways are protein kinase cascades that play key roles in the regulation of seed germination [14,15]. The overexpression of *CsNMAPK* in tobacco enhances seed germination under stress conditions [16], and the *Arabidopsis* MAPK phosphatase PP2C5 affects seed germination [17]. Reactive oxygen species (ROS) induce a MAPK-dependent regulation of ABA and GA levels to affect the resumption of metabolism during seed germination [18,19]. However, the precise signaling responses in seed germination of *P*. *polyphylla* are not yet fully understood.

It is necessary to detect the potential regulators of seed germination to obtain clues about how to improve the artificial cultivation of *P*. *polyphylla*. In this study, we analyzed the dynamic transcriptomes of *P*. *polyphylla* seeds at three germination stages using RNA sequencing (RNA-seq) technology. The differentially expressed genes associated with carbohydrate metabolism, lipid metabolism, signal transduction, and translation were highlighted in this study. The identified genes provide a valuable resource for studying the molecular basis of seed germination in *P. polyphylla*.

## 2. Results

### 2.1. Illumina Sequencing and De Novo Assembly

**Figure 1:**
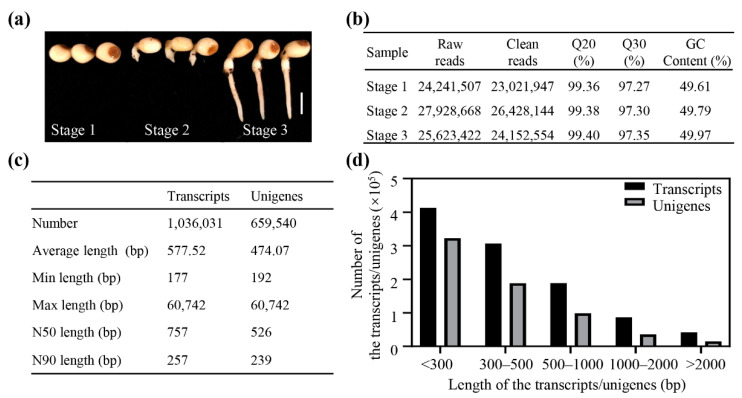
Summary of the transcriptome sequencing. (**a**) Seeds were harvested at three germination stages for RNA-seq. Bar is 0.5 cm. (**b**) Summary data of RNA-seq. (**c**) Number and length of the transcripts and unigenes. (**d**) Distributions of the transcripts and unigenes.

To investigate the potential genes involved in seed germination of *P*. *polyphylla*, three cDNA libraries were constructed from mRNA of three different germination stages (stage 1, stage 2, and stage 3) (Figure 1a) and then were sequenced using the Illumina Nova instrument. After removing the adapters, low-quality data, and ambiguous reads, we obtained approximately 23, 26, and 24 million clean reads in seeds of stage 1, stage 2, and stage 3, respectively (Figure 1b). The Phred quality score (Q20 and Q30) in each sample was greater than 95%, and the GC percentage in each sample was approximately 50%. Next, the clean reads were used to assemble the transcriptome data using the Trinity software. Based on overlapping information in high-quality reads, 1,036,031 transcripts and 659,540 unigenes were generated (Figure 1c). N50 and N90 (N50 and N90’s size was computed by sorting all transcripts from largest to smallest and by determining the minimum set of transcripts whose sizes total 50% and 90% of the entire genome, respectively) are 757 and 257 bp for the transcripts, respectively, and 526 and 239 bp for the unigenes. The length distributions of the transcripts and unigenes are shown in Figure 1d.

### 2.2. Functional Classification of the Unigenes

**Figure 2:**
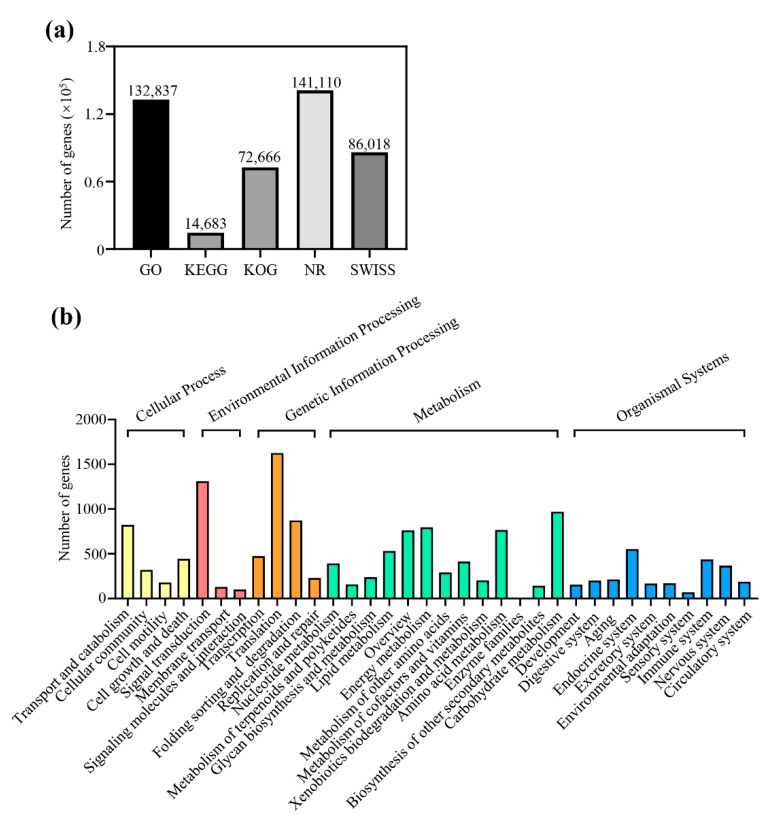
Functional classification of the unigenes. (**a**) The number of annotated unigenes in five public databases. (**b**) KEGG classifications of unigenes.

For functional annotation, all the sequences in the public databases, including NCBI non-redundant protein sequences (NR), Swiss-Prot, euKaryotic Ortholog Groups (KOG), Gene ontology (GO), and Kyoto Encyclopedia of Genes and Genomes (KEGG), were used to annotate the assembled unigenes. A total of 447,314 unigenes were successfully matched with the searched databases; however, 32.16% of the unigenes were unmatched (Figure 2a). It is possible that the genome and EST information is insufficient for *P. polyphylla*. The bioinformatics technical limitations, including sequencing depth and read length, are another possible reason. To further reveal the potential functions of the transcripts, the functional classification of the unigenes was performed using KEGG assignments. It showed that the unigenes were clustered into 34 functional pathways, of which 4 were cellular processes, 3 were environmental information processing, 4 were genetic information processing, 13 were metabolism, and 10 were organismal systems (Figure 2b).

### 2.3. Identification and Functional Classification of DEGs

**Figure 3:**
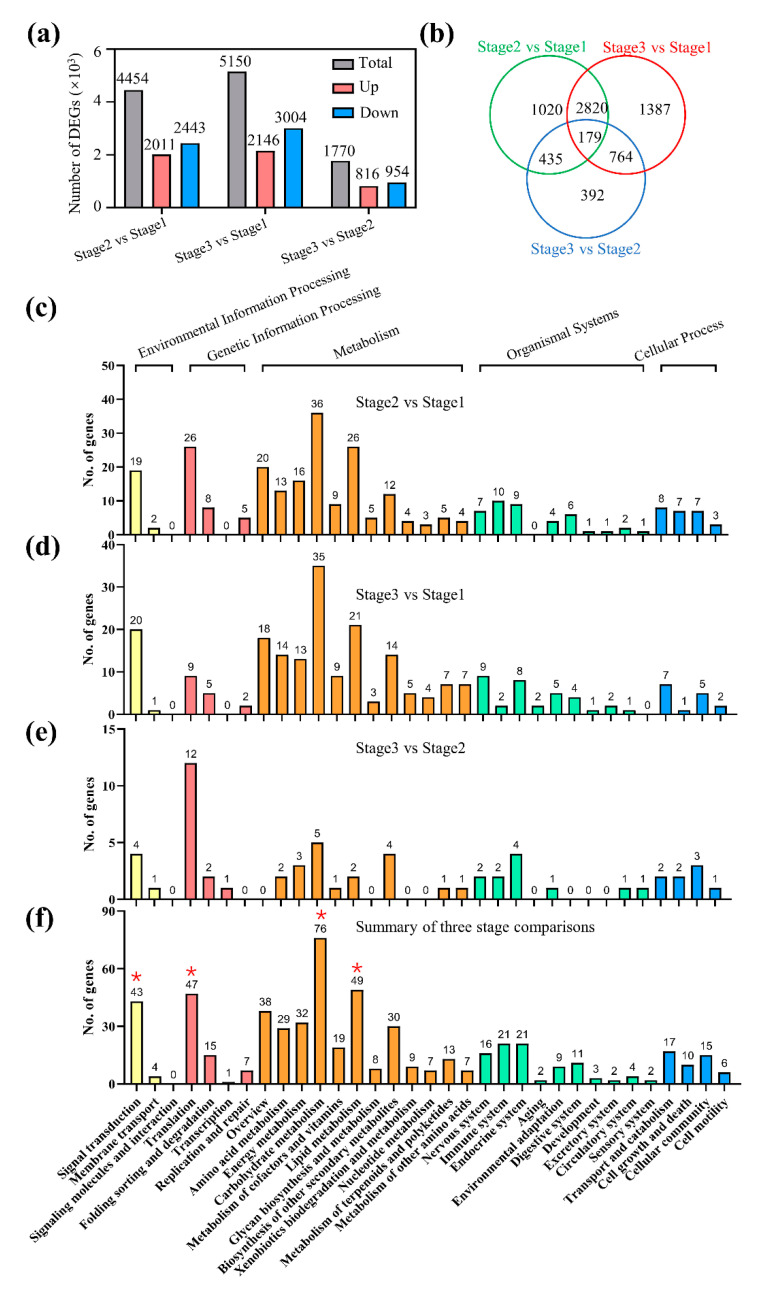
KEGG enrichment of differentially expressed genes. (**a**,**b**) Summary of differentially expressed genes at stage 1, stage 2, and stage 3. KEGG pathways enrichment in (**c**) stage 1 to stage 2, (**d**) stage 1 to stage 3, (**e**) stage 2 to stage 3, and (**f**) three germination stages. * means the genes were focused on in the following study.

Differentially expressed genes (DEGs) were identified based on the Reads Per Kilobase per Million mapped reads (RPKM) ≥ 5 in at least one sample, log_2_ fold change≥ 1, and adjusted *p* values (*q*) < 0.001. According to the standard, 4454 DEGs were identified between stage 1 and stage 2, of them 2011 up-regulated and 2433 down-regulated (Figure 3a; Table S1). From stage 1 to stage 3, a total of 5150 DEGs were detected, of which 2146 were up-regulated and 3004 were down-regulated. Meanwhile, a total of 1770 DEGs were identified from stage 2 to stage 3, of which 816 were up-regulated and 954 were down-regulated. By comparison, 1020, 392, and 1387 DEGs were specifically detected in stage 1 to stage 2, stage 2 to stage 3, and stage 1 to stage 3, respectively (Figure 3b). A total of 614, 2999, and 943 DEGs were simultaneously identified in both stage 1 to stage 2 and stage 2 to stage 3, stage 1 to stage 2 and stage 1 to stage 3, and stage 1 to stage 3 and stage 2 to stage 3, respectively. Interestingly, 179 DEGs were simultaneously detected in three stages. KEGG enrichment revealed that these DEGs were involved in environmental information processing, genetic information processing, the metabolism of organismal systems, and cellular processes (Figure 3d–f). Of them, the most significantly enriched pathways were carbohydrate metabolism, lipid metabolism, signal transduction, and translation at three germination stages (Figure 3f). Thus, the DEGs involved in carbohydrate metabolism, lipid metabolism, signal transduction, and translation were focused on in the following study.

### 2.4. DEGs Involved in Carbohydrate Metabolism

**Figure 4:**
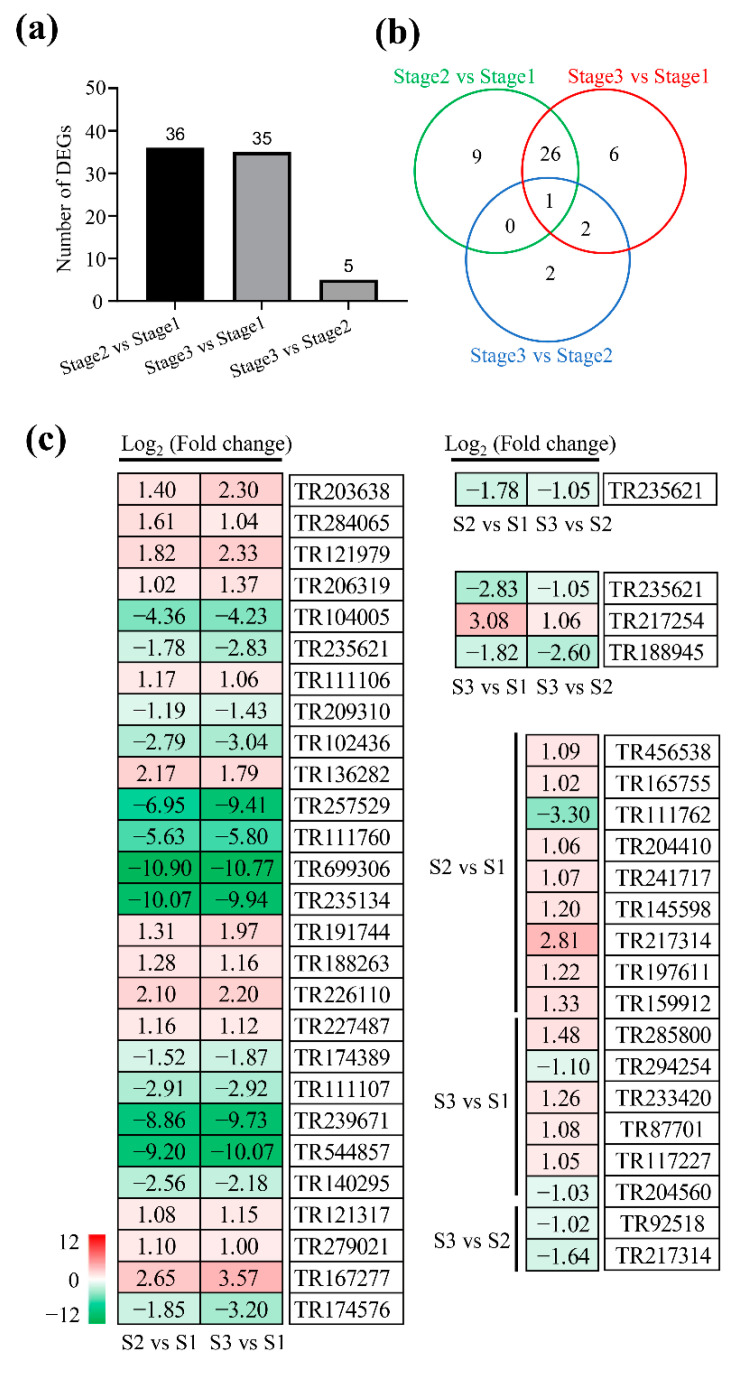
Analysis of differentially expressed genes involved in carbohydrate metabolism. (**a**,**b**) Summary of differentially expressed genes at stage 1, stage 2, and stage 3. (**c**) The list of differentially expressed genes. Red and green represent up-regulated and down-regulated, respectively.

A total of 36, 5, and 35 DEGs related to carbohydrate metabolism were identified at stage 1 to stage 2, stage 2 to stage 3, and stage 1 to stage 3, respectively (Figure 4a; Table S2). By comparison, 9, 6, and 2 DEGs were specifically detected at stage 1 to stage 2, stage 1 to stage 3, and stage 2 to stage 3, respectively (Figure 4b). However, 1 DEG was involved in three stages, and 27, 1, and 3 DEGs were simultaneously identified in both stage 1 to stage 2 and stage 1 to stage 3, stage 1 to stage 2 and stage 2 to stage 3, and stage 2 to stage 3 and stage 1 to stage 3, respectively (Figure 4b). For example, the expression of gene encoding glutamate decarboxylase (TR235621) was significantly decreased at three stages, while beta-amylase 3 (TR217254) was significantly increased in both stage 1 to stage 3 and stage 2 to stage 3 (Figure 4b). The expression of gene encoding alpha-amylase isozyme 3D isoform X1 (TR167277) was significantly increased in both stage 1 to stage 2 and stage 1 to stage 3, while glutamine synthetase (TR111760) and malate dehydrogenase (TR174389) were significantly decreased (Figure 4c).

### 2.5. DEGs Involved in Lipid Metabolism

**Figure 5:**
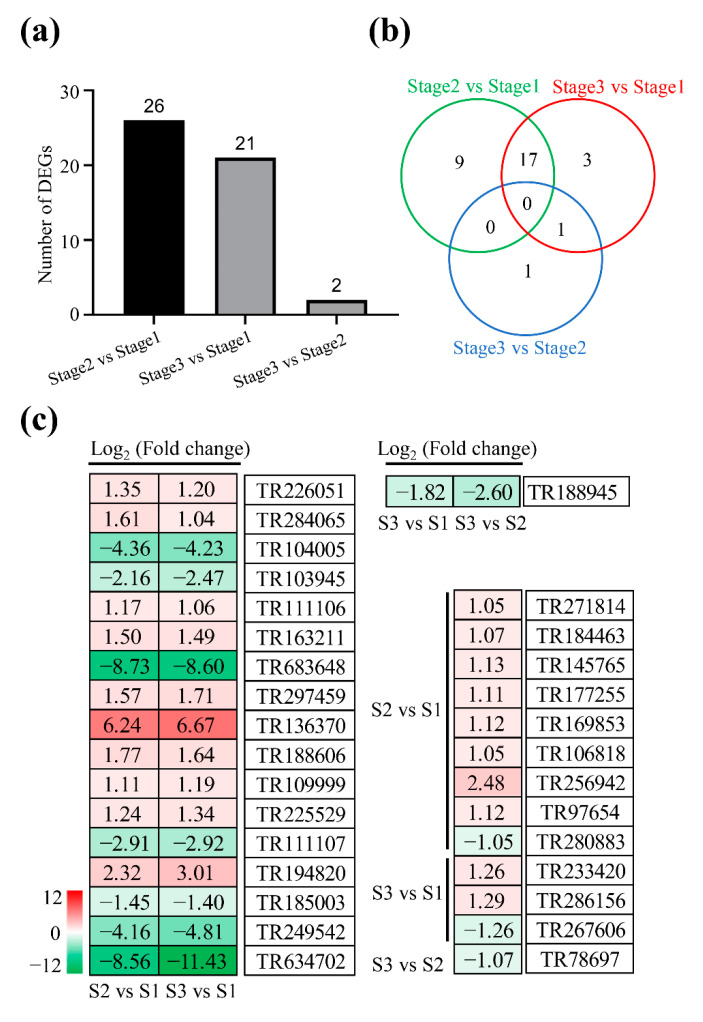
Analysis of differentially expressed genes involved in lipid metabolism. (**a**,**b**) Summary of differentially expressed genes at stage 1, stage 2, and stage 3. (**c**) The list of differentially expressed genes. Red and green represent up-regulated and down-regulated, respectively.

A total of 26, 2, and 21 DEGs related to lipid metabolism were identified at stage 1 to stage 2, stage 2 to stage 3, and stage 1 to stage 3, respectively (Figure 5a; Table S3). By comparison, 9, 3, and 1 DEGs were specifically detected at stage 1 to stage 2, stage 1 to stage 3, and stage 2 to stage 3, respectively (Figure 5b). However, 17 and 1 DEGs were simultaneously identified in both stage 1 to stage 2 and stage 1 to stage 3, and stage 2 to stage 3 and stage 1 to stage 3, respectively. For example, the expressions of genes encoding glycerol-3-phosphate acyltransferase 5 (TR226051), 3-ketoacyl-CoA synthase 12-like (TR163211), phospholipase D alpha 1 (TR297459), allene oxide cyclase (TR188606), and long-chain acyl-CoA synthetase 2 (TR225529) were significantly up-regulated, while alpha-galactosidase (TR111107) was significantly down-regulated in both stage 1 to stage 2 and stage 1 to stage 3 (Figure 5c).

### 2.6. DEGs Involved in Signal Transduction

**Figure 6:**
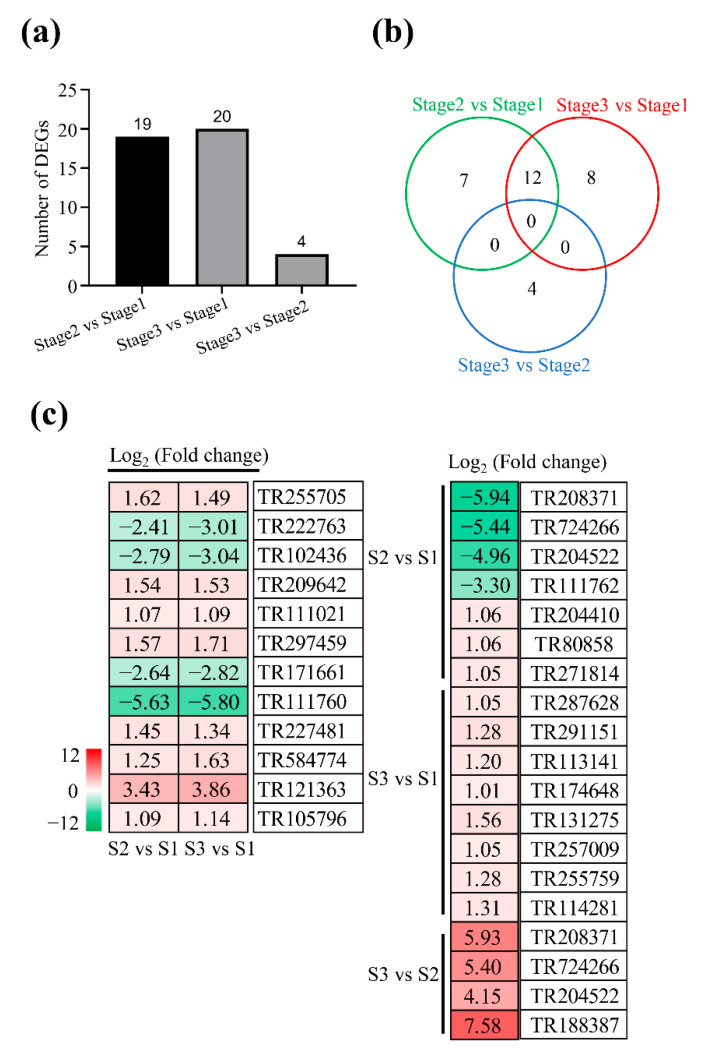
Analysis of differentially expressed genes involved in signal transduction. (**a**,**b**) Summary of differentially expressed genes at stage 1, stage 2, and stage 3. (**c**) The list of differentially expressed genes. Red and green represent up-regulated and down-regulated, respectively.

A total of 19, 4, and 20 DEGs related to signal transduction were identified at stage 1 to stage 2, stage 2 to stage 3, and stage 1 to stage 3, respectively (Figure 6a; Table S4). By comparison, 7, 8, and 4 DEGs were specifically detected in stage 1 to stage 2, stage 1 to stage 3, and stage 2 to stage 3, respectively (Figure 6b). However, 12 DEGs were simultaneously identified in both stage 1 to stage 2 and stage 1 to stage 3. These DEGs were mostly involved in the plant hormone signal transduction and the MAPK signaling pathway during seed germination. For example, the expressions of genes encoding auxin-responsive protein IAA (TR227481) and mitogen-activated protein kinase kinase 9 (TR111021) were significantly increased in both stage 1 to stage 2 and stage 1 to stage 3, while the expression of ABA-responsive element binding factor (TR222763) was significantly decreased (Figure 6c).

### 2.7. DEGs Involved in Translation

**Figure 7:**
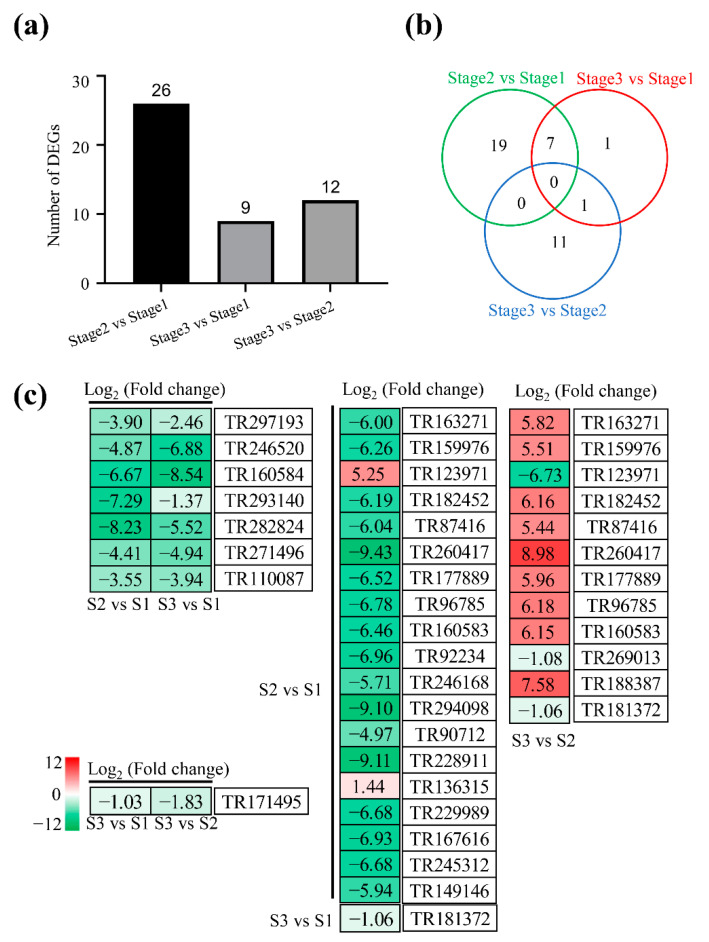
Analysis of differentially expressed genes involved in translation. (**a**,**b**) Summary of differentially expressed genes at stage 1, stage 2, and stage 3. (**c**) The list of differentially expressed genes. Red and green represent up-regulated and down-regulated, respectively.

A total of 26, 12, and 9 DEGs related to translation were identified at stage 1 to stage 2, stage 2 to stage 3, and stage 1 to stage 3, respectively (Figure 7a; Table S5). By comparison, 19, 1, and 11 DEGs were specifically detected at stage 1 to stage 2, stage 1 to stage 3, and stage 2 to stage 3, respectively (Figure 7b). However, 7 and 1 DEGs were simultaneously identified in both stage 1 to stage 2 and stage 1 to stage 3, and stage 2 to stage 3 and stage 1 to stage 3, respectively. For example, the expressions of genes encoding small and large subunit ribosomal proteins (TR297193, TR160584, TR293140, TR282824, TR271496, and TR110087) were significantly decreased in both stage 1 to stage 2 and stage 1 to stage 3. Similarly, the expressions of genes encoding small and large subunit ribosomal proteins (TR163271, TR159976, TR123971, TR182452, TR87416, TR260417, TR96785, and TR160583) were significantly decreased in both stage 1 to stage 2 and stage 2 to stage 3 (Figure 7c).

### 2.8. Validation of DEGs by qRT-PCR

**Figure 8:**
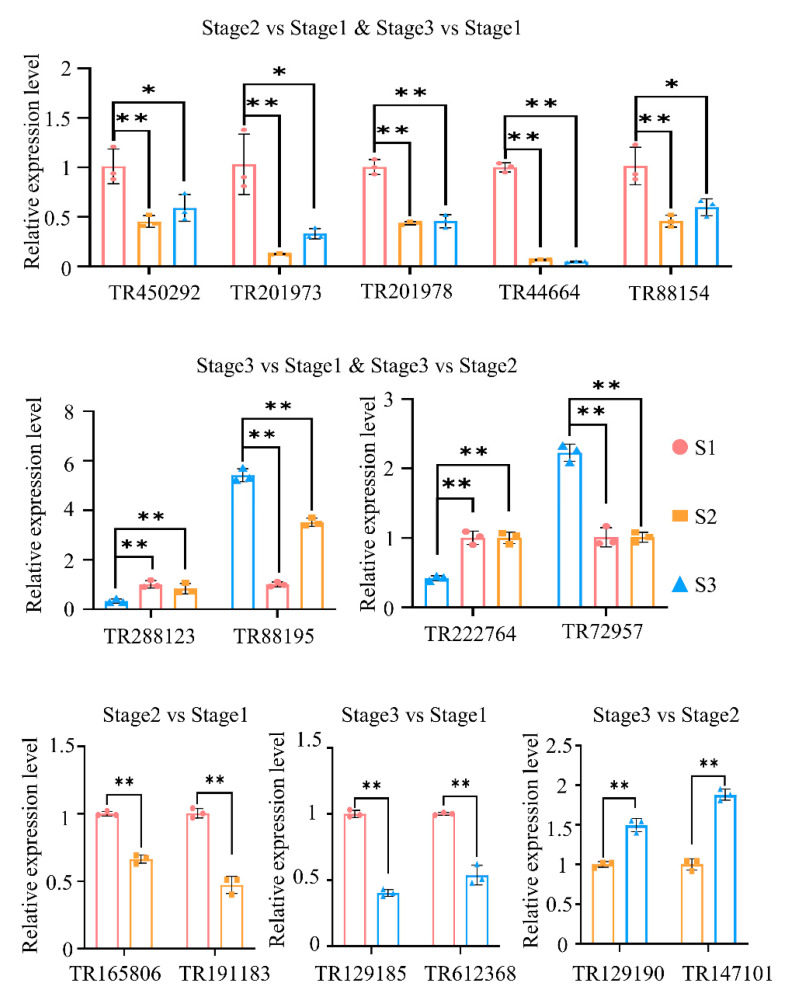
The relative expression levels of the randomly selected differentially expressed genes detected using qRT-PCR. The *Actin* gene was used as an internal control. S1, S2, and S3 mean stage 1, stage 2, and stage 3, respectively. * and ** means significant difference at the 5% and 1% level using Student’s *t*-test, respectively.

To validate the reliability of RNA-seq results, several randomly selected DEGs were conducted for expression qualification using the qRT-PCR approach. The expression patterns of these genes were consistent with the results of transcriptome data (Figure 8). For example, the expressions of genes TR450292, TR201973, TR201978, TR44664, and TR88154 simultaneously identified in both stage 1 to stage 2 and stage 1 to stage 3 were significantly decreased. The expressions of genes TR288123 and TR222764 were significantly increased in both stage 1 to stage 2 and stage 2 to stage 3, while the genes TR88195 and TR72957 were significantly decreased.

## 3. Discussion

Seeds of *P. polyphylla* have morphophysiological dormancy [3]. The speed of germination is an important trait for the artificial cultivation of *P. polyphylla*. The annual average temperature is approximately 20 °C in Kunming of Yunnan province. Thus, here, the warm stratification at 20 °C for 90 days was conducted in *P. polyphylla* to obtain seeds with different germination states. Finally, the seeds at three germinated states, including ungerminated seeds (stage 1) (approximately 65%), germinated seeds with a 0.5 cm radicle length (stage 2) (approximately 20%), and germinated seeds with a 2.0 cm radicle length (stage 3) (approximately 5%), were harvested to detect the potential regulators of seed germination in *P. polyphylla*. We observed that more DEGs existed in stage 1 to stage 2 and stage 1 to stage 3 than that in stage 2 to stage 3, suggesting that active changes occurred in the early and middle germination stages in *P. polyphylla*. Further KEGG analysis showed that the most abundant DEGs were associated with carbohydrate metabolism, lipid metabolism, signal transduction, and translation. Thus, the details of DEGs involved in carbohydrate metabolism, lipid metabolism, signal transduction, and translation were focused on in this study.

The mobilization of reserves plays an important role in early seed germination and seedling growth in plants. It is well-known that α-amylase and β-amylase play crucial roles in starch metabolism during seed germination [20,21]. Similarly, the induced expressions of genes encoding alpha-amylase isozyme and beta-amylase 3 were observed during the seed germination of *P. polyphylla* in this study. Interestingly, we found that several genes associated with amino acid metabolism, such as glutamate decarboxylase and glutamine synthetase, were differentially expressed during seed germination in *P. polyphylla*. Glutamate decarboxylase is an enzyme that catalyzes the production of γ-amino butyric acid (GABA) from glutamate through a decarboxylation reaction. The accumulation of GABA has been reported to be involved in seed germination and seedling growth in soybean and *B. juncea* [22,23]. The glutamine synthetase cycle is involved in amino acid metabolism during germination and post-germinative growth in Medicago truncatula [24]. It would be interesting to determine whether glutamate decarboxylase and glutamine synthetase have the potential roles of germination regulation via influencing amino acids. Moreover, we observed that the gene encoding alpha-galactosidase was involved in both carbohydrate and lipid metabolism. It has been revealed that alpha-galactosidase is involved in desiccation tolerance during seed maturation and acts as a source of stored energy utilized by germinating seeds [25]. Thus, we speculated that alpha-galactosidase might be involved in the potential regulation of seed germination by affecting seed maturation in *P. polyphylla*.

Previously, a total of 62 genes associated with hormone signaling pathways, such as ABA receptors PYR/PYL/RCAR, ABA signaling protein type 2C phosphatases (PP2Cs), SUCROSE NONFERMENTING1-RELATED SUBFAMILY2 (SnRK2), DELLA protein gene, auxin influx genes (AUX1 and LAX3), and IAA family genes, were identified in seeds during a warm stratification in *P. polyphylla* [5]. Similarly, several genes, such as ABA-responsive element binding factor (ABF) and auxin-responsive protein (IAA30) involved in hormone signaling pathways were identified during seed germination in *P. polyphylla* in this study. We observed that the *ABF* was significantly down-regulated in both stage 1 to stage 2 and stage 1 to stage 3, while the *IAA30* was significantly induced. ABF is a key component of the ABA signaling pathway and is used to control gene expression [26]. It has been indicated that the key protein kinases SnRK2.2 and SnRK2.3 regulate seed germination through the phosphorylation of ABFs in *Arabidopsis* [27]. The loss-of-function mutant of auxin-responsive protein gene *IAA8* delays seed germination in *Arabidopsis*, and *IAA8* enhances seed germination through the inhibition of *AtABI3* expression [28]. Thus, we speculated that the down-regulation of *ABF* and up-regulation of *IAA30* might contribute to seed germination by influencing the ABA signaling pathway in *P. polyphylla*.

The MAPK cascades are key signaling modules for responding to ABA during seed germination [29]. For example, the *Arabidopsis* MAPK phosphatase PP2C5 affects seed germination, stomatal aperture, and ABA-inducible gene expression [17]. Similarly, we observed that the protein phosphatase 2C (PP2C) was involved in both hormone and MAPK signaling pathways during the seed germination of *P. polyphylla*. We speculated that PP2C might be involved in seed germination through the ABA signaling pathway. The tomato *MAPK11* regulates seed germination and ABA signaling by phosphorylating SnRKs [30]. The loss-of-function mutant of barley *MAPK6* causes abnormal embryo development, which leads to lessened seed germination and shootless seedlings [31]. In this study, we observed that the expressions of *MAPK9* and *MAPK18* were significantly increased during seed germination in *P. polyphylla*. It would be interesting to reveal whether *MAPK9* and *MAPK18* regulate seed germination via the crosstalk of ABA and MAPK signaling pathways in the future.

The hormone ABA is a key signal regulating seed dormancy and seed germination. In this study, the identified potential regulators of seed germination, such as ABF, IAA30, PP2C, MAPK9, and MAPK18, might be involved in the ABA signaling pathway. Moreover, we identified the potential regulators of seed germination, such as glutamate decarboxylase and glutamine synthetase, that might be involved in amino acid metabolism. Furthermore, a larger number of DEGs encoding small and large subunit ribosomal proteins involved in translation [32,33] were also identified during seed germination in *P. polyphylla*. We will focus on the functional analysis of these genes to reveal the molecular mechanism of seed germination in *P. polyphylla* in the future.

## 4. Materials and Methods

### 4.1. Plant Materials and Seed Germination

Freshly matured fruits of *P. polyphylla var. yunnanensis* were collected in Yunnan Province of China in October 2019. The freshly collected seeds were placed into moistened sand for seed germination at 20 °C for 90 days. Three states of seeds, including ungerminated seeds (stage 1), germinated seeds with 0.5 cm radicel length (stage 2), and germinated seeds with 2.0 cm radicel length (stage 3), were observed after 90 days. The seeds at three different states were harvested, respectively, for RNA sequencing. Three biological replications were performed.

### 4.2. RNA Sequencing Approach

Ten seeds per replication were used for RNA extraction in each sample. Total RNA was isolated separately from each sample using a Total RNA Rapid Extraction Kit (BioTeke Corporation, Wuxi, China). Three biological replications were performed. Ribosomal RNA (rRNA) in the samples was depleted using the Ribo-Zero™ Magnetic Kit (Epicentre, Madison, WI, USA) according to the manufacturer’s instructions. The cDNA libraries were generated and sequenced by Shanghai Hanyu Biotechnology Company Ltd. (Shanghai, China) using an Illumina sequencing NovaSeq platform.

### 4.3. Transcriptome Assembly and Functional Annotation

Clean reads were obtained after removing the adaptor sequences, unknown sequences, and low-quality reads using Trimmomatic v0.32 [34]. Sequence quality was evaluated with Fastqc v0.10.0 (http://www.bioinformatics.babraham.ac.uk/projects/fastqc; accessed on 30 Feburary 2020). Transcriptome de novo assembly was generated by using Trinity v2.06, the min_kmer_cov was set to 2, and all other parameters were set to default [35]. To annotate the assembled transcripts, the unigene sequences were aligned against the databases of NR, Swiss-Prot, KOG, GO, and KEGG using Blastp v2.2.22 [36] with a significance threshold of E-value < 10^−5^.

### 4.4. Differentially Expressed Genes Analysis

Differentially expressed genes (DEGs) were determined using DEGseq v1.20.0 with the MARS (MA-plot-based method with random sampling model) method [37]. Adjusted *p* values (*q*) were generated using the Benjamini and Hochberg correction to control the false discovery rate (FDR). In this analysis, the genes with a threshold *q*-value < 0.001, log_2_ fold change ≥1, and RPKM ≥ 5 in at least one sample were identified as differentially expressed.

### 4.5. Quantitative Real-Time PCR

Approximately 4 μg of total RNA was reverse-transcribed to cDNA using the HiScript^®^ II Reverse Transcriptase system (Vazyme Biotech Co., Ltd., Nanjing, China). The quantitative real-time PCR (qRT-PCR) was performed in 20 μL reaction mixture with the CFX96 Real-Time System (Bio-Rad, Hercules, CA, USA). The PCR conditions were as follows: 95 °C for 2 min, followed by 40 cycles of 95 °C for 5 s and 60 °C for 10 s. The *Actin* gene was used as an internal control. The primers were designed using Primer Premier 5 and listed in Table S6. The levels of gene expression were calculated by using the comparative 2^−ΔΔC^_T_ value method [38].

## 5. Conclusions

In summary, a comprehensive transcriptome was performed to reveal the potential regulators of seed germination in *P. polyphylla* in this study. We observed that the DEGs associated with carbohydrate metabolism, lipid metabolism, signal transduction, and translation might play important roles in the seed germination of *P. polyphylla*. The identified genes provide a valuable resource to investigate the molecular mechanism of seed germination of *P. polyphylla* in the future.

## Data Availability

Not applicable.

## Supplementary Materials

The following supporting information can be downloaded at: https://www.mdpi.com/article/10.3390/plants11182400/s1, Table S1: Differentially expressed genes during seed germination; Table S2: Differentially expressed genes related to carbohydrate metabolism; Table S3: Differentially expressed genes related to lipid metabolism; Table S4: Differentially expressed genes related to signal transduction; Table S5: Differentially expressed genes related to translation; Table S6: List of primer pairs used in this study.
